# Improving health equity for ethnic minority women in Thai Nguyen, Vietnam: qualitative results from an mHealth intervention targeting maternal and infant health service access

**DOI:** 10.1093/pubmed/fdy165

**Published:** 2018-09-25

**Authors:** B McBride, J D O’Neil, Trinh T Hue, R Eni, C Vu Nguyen, L T Nguyen

**Affiliations:** 1 School of Population and Public Health, University of British Columbia, Canada; 2 Faculty of Health Sciences, Simon Fraser University, Burnaby, Canada; 3 Institute of Population, Health and Development, Hanoi, Vietnam; 4 Nossal Institute for Global Health, University of Melbourne, Australia

**Keywords:** behaviour change communication, eHealth, ethnic minorities, health equity, mHealth, MNCH, mobile health, Vietnam

## Abstract

**Background:**

Ethnic minority women (EMW) in Vietnam experience disproportionately high infant and maternal mortality rates due to low social status, poverty and remoteness from health centres. This project piloted and evaluated a low-cost mobile health (mHealth) intervention called mMom utilizing behaviour change communication (BCC) to improve access to maternal, newborn and child health (MNCH) services and health equity among EMW living in remote areas.

**Methods:**

The mMom intervention built an integrated mHealth platform which sent timely MNCH information and BCC text messages to participants, and engaged health workers towards increasing their interaction and building demand for quality natal care. Mid-term and final qualitative evaluations were conducted to assess the intervention’s acceptability and impact.

**Results:**

In evaluations, all participants expressed satisfaction with the quality, timeliness and convenience of the messages, and health workers reported increased efficiency and quality of care. The use of BCC increased care-seeking from EMW and strengthened relationships with health providers.

**Conclusion:**

The mMom project demonstrated the acceptability of mHealth in a remote Vietnamese region with a high proportion of disadvantaged EMW. The messages promoted increased contact between participants and health providers, which holds potential to address the marginalization of EMW from the health system.

## Resumen

**Contexto:**

Las mujeres de Vietnam que pertenecen a minorías étnicas presentan tasas de mortalidad materna e infantil desproporcionadamente altas debido al bajo nivel social, la pobreza y la lejanía de los centros de salud. Este proyecto llevó a cabo y evaluó una intervención de salud móvil de bajo costo llamada mMom, que utilizaba estrategias de comunicación para cambios en el comportamiento con el fin de mejorar el acceso a los servicios de salud materna, neonatal e infantil, así como para alcanzar la equidad en materia de salud entre las mujeres de minorías étnicas que viven en zonas remotas.

**Métodos:**

La intervención mMom creó una plataforma integrada de salud móvil que enviaba a las participantes información sobre salud materna, neonatal e infantil, y mensajes de texto relativos a la comunicación para cambios en el comportamiento. Además, esta plataforma incluyó a los profesionales de la salud con el objetivo de aumentar su interacción y fomentar la demanda de atención neonatal de calidad. Se realizaron evaluaciones cualitativas intermedias y finales para evaluar la aceptabilidad y el impacto de la intervención.

**Resultados:**

En las evaluaciones, todas las participantes expresaron estar satisfechas con la calidad, la prontitud y la conveniencia de los mensajes, y los profesionales de la salud manifestaron que aumentaron la eficacia y la calidad de la atención. El uso de las comunicaciones para cambios en el comportamiento aumentó la cantidad de consultas por parte de mujeres de minorías étnicas y fortaleció su relación con los profesionales de la salud.

**Conclusión:**

El proyecto mMom demostró la aceptabilidad de la salud móvil en una región remota de Vietnam, con una alta proporción de mujeres de minorías étnicas desfavorecidas. Los mensajes promovieron un mayor contacto entre las participantes y los profesionales de la salud, lo cual tiene el potencial de resolver la marginalización de estas mujeres del sistema de salud.

**Palabras clave:**

comunicaciones para cambios en el comportamiento, cibersalud, minorías étnicas, igualdad en materia de salud, salud móvil, atención materna, neonatal e infantil, Vietnam

## RÉSUMÉ

**Contexte:**

Les femmes issues de minorités ethniques au Vietnam affichent des taux de mortalité infantile et maternelle démesurément élevés en raison de leur statut social peu élevé et de l’éloignement des centres de santé. Ce projet a dirigé et évalué une intervention peu coûteuse de santé mobile appelée mMom faisant appel à une communication axée sur la modification des comportements pour améliorer l’accès aux services de santé destinés aux mères, aux nouveau-nés et aux enfants (SMNE) et à une équité en matière de santé chez les femmes issues de minorités ethniques qui habitent dans des régions éloignées.

**Méthodes:**

L’intervention mMom a mis en place une plateforme de santé mobile qui envoyait aux participantes en temps opportun des renseignements sur les services de SMNE et des messages textes de communication axée sur la modification des comportements, et mobilisait les travailleurs de la santé en vue d’accroître les échanges et créer une demande pour des soins aux nouveau-nés de qualité. Des évaluations qualitatives ont été réalisées en milieu et en fin de parcours pour évaluer la façon dont les interventions avaient été acceptées et leur incidence.

**Résultats:**

Dans les évaluations, toutes les participantes ont exprimé leur satisfaction au sujet de la qualité et du caractère opportun et pratique des messages, et les travailleurs de la santé ont fait part d’une efficacité et d’une qualité accrues des soins. Le recours à une communication axée sur la modification des comportements a encouragé davantage les femmes issues de minorités ethniques à aller chercher des soins, et a renforcé le lien avec les fournisseurs de soins de santé.

**Conclusion:**

Le projet mMom a montré que la santé mobile est acceptée par une grande partie de femmes issues de minorités ethniques défavorisées dans une région éloignée du Vietnam. Les messages ont favorisé un accroissement des contacts entre les participantes et les fournisseurs de soins de la santé, ce qui peut contribuer à remédier à la marginalisation des femmes issues de minorités ethniques.

**Mots clés:**

communication axée sur la modification de comportements, cybersanté, minorités ethniques, équité en matière de santé, santé mobile, SMNE, Vietnam

## Background

### Ethnic minority health in Vietnam

Vietnam’s remarkable progress on child mortality, maternal health and gender equality indicators over the past 3 decades has largely excluded ethnic minority (EM) groups. While the nation’s under-five mortality and maternal mortality rates have halved since 1990,^1,2^ disaggregated data from the last three Vietnam Censuses show that EM people have significantly higher total fertility, infant and child mortality rates compared to the Kinh (ethnic majority), and are less likely to attend maternal care.^3^ Under-nutrition and stunting rates amongst children in mountainous areas, where ethnic minority populations are concentrated, are three times higher than rates in the wealthier lowland provinces.^4^ Maternal mortality rates were also found to be significantly higher among EM women (EMW) compared to Kinh women, at 316 versus 81 per 100 000 births, respectively.^4^ In 2011, 55% of EMW in Vietnam reported having their most recent birth in a health facility, in contrast to over 95% of Kinh women.^5^ These disparities are similar across all regions in Vietnam with large EM populations.

Decades of structural deprivation and social exclusion have resulted in poor education, low socio-economic and class status, and rural residence for EM peoples in Vietnam, which are primary determinants of their relatively poor health outcomes.^6^ Despite Vietnam’s swift economic development, wealth disparities between Kinh and EM groups have remained nearly constant between 2006 and 2014.^7^ Limited access to information, low reproductive health knowledge, poor maternal, newborn and child health (MNCH) behaviours and poor access to health services are key factors contributing to disproportionately poor MNCH outcomes among EMW and their newborns.^6,8^ In addition, there are fewer and lower capacity health facilities and staff in remote regions.^8^

### Global mHealth for MNCH

The resistance of health equity challenges to traditional development approaches and the near-worldwide penetration of inexpensive mobile phone service have contributed to a global burgeoning of mHealth initiatives, and mHealth has been widely applied to maternal and infant health in LMICs. A 2016 systematic review of maternal mHealth projects found that all interventions on maternal service utilization showed significant increases in accessing MNCH health care services, particularly in antenatal and postnatal care attendance.^9^ Studies by Lund *et al.*^10,11^ in Zanzibar, Kaewkungwal *et al.*^12^ near the Thai-Myanmar border and Odeny *et al.*^13^ in Kenya all report positive results for improved antenatal care associated with mHealth interventions. mHealth can also support healthy MNCH behaviours, as studies have identified positive outcomes in the areas of infant feeding, post-abortion contraception initiation, health service utilization, and immunization in China,^14^ Malawi,^15^ Cambodia,^16^ Nigeria^17^ and Bangladesh.^18^ Research also indicates mHealth interventions can increase staff efficiency and improve data entry and monitoring.^19–21^

Despite these advances, certain areas remain underdeveloped. The majority of current mHealth programs apply a passive IEC approach, while few have utilized active behaviour change communication (BCC) or aimed to improve interaction between the target population and health workers:^22^ a particular concern for minority groups historically underserved by formal health systems. In addition, few studies have applied a health equity lens to consider how mHealth may mitigate physical or social facets of marginalization, such as proximity to health centres in remote communities, low confidence in care-seeking due to lack of information or satisfaction with available care. Through its research design, this intervention (the mMom project) aimed to utilize BCC, emphasize interaction between women and CHWs, and evaluate women’s confidence and readiness to access health care.

## Methodology

The mMom project was developed by the Institute for Population, Health and Development in Hanoi (PHAD) and Simon Fraser University (SFU) in Vancouver and funded by the International Development Research Centre Canada (IDRC). The project aimed to determine whether integrated use of an electronic health information system, low-cost mobile technology and a user-provider interaction model could improve access to MNCH care amongst EMW and their newborns. In brief, the mMom software and its operation was developed by Vietnam electronic health (eHealth) Medical Investment and Communication, in consultation with the provincial Ministry of Health. mMOM is a new mHealth component, aimed at health communication, as part of the existing HMIS for maternal and child health in the Thai Nguyen Province. However, the system can also function independently and can be applied to other modules of the HMIS. The mMom database is able to use each patient’s unique identity code as established in the HMIS. The platform aims to coordinate and add support to commune health workers’ existing tasks in monitoring pregnancy and new motherhood in community women. A more detailed description of the project’s development is available elsewhere.^23^

### Intervention development

The project design required the identification of one district within a mountainous northern province where a substantial proportion of the population are EMW. In discussions with national Ministry of Health (MOH) representatives, Thai Nguyen province in northern Vietnam was selected because EM constitute 27% of a population of 1.3 million^24^ where the majority live in remote areas of the province, active engagement of the Thai Nguyen Provincial Health Department (TNHD) in developing an electronic Health Management Information System (HMIS), and pre-existing and strong collaborations between PHAD and TNHD. All hospitals and 181 communes in nine districts are fully integrated into an electronic record management system, and 100% of commune health centres are connected by high speed Internet to district and provincial health centres.^23^

In discussions with TNHD officials, Dinh Hoa district was selected for the pilot intervention because it was one of two mountainous districts where most of the population are EM. It also reflected the general social and demographic characteristics of other remote districts in northern Vietnam and yet was reasonably accessible to the research team.

Prior to the intervention’s development, rapid ethnographic fieldwork was conducted in Dinh Hoa district. This fieldwork assessed the extent of mobile phone use; literacy barriers; socio-cultural influences on phone ownership and use; MNCH indicators; health service utilization and health worker capacity. In Dinh Hoa, maternal health information, education and communication (IEC) is largely delivered through posters, leaflets and quarterly group counselling sessions conducted by commune health workers (CHWs) at health centres. These sessions provide general information to some women at all stages of pregnancy. In-depth interviews and several focus group discussions with 8–10 participants each, CHWs and local women reported that this traditional IEC approach was not comprehensive or timely. Women commented that the counselling sessions lacked specificity and detail, and that it was challenging to absorb sufficient information to benefit for the next few months of pregnancy. The sessions were structured to provide unidirectional IEC delivery, allowing few opportunities for deeper interaction between a woman and her CHW. CHWs also reported that the posters and leaflets lacked detail, and that women did not always know how to apply information from them. In summary, the strategy of providing generalized, unidirectional IEC to all pregnant community women was found to be limited in its ability to support maternal health for EMW.

All communes in Dinh Hoa district were stratified into centrally located, middle and remote communes, and then random selection was applied to choose eight project communes and four control communes in the district. The mMom intervention target population were all women living in the selected communes who had a current pregnancy, a new pregnancy in the first 2 years of the project intervention, or who had a child under 1 year of age.

Participants (intervention users) were recruited at commune health centres by CHWs, and upon providing informed consent, would be registered and begin to receive messages based on their estimated week or pregnancy or their infant’s age. Women from four non-project communes, also recruited at commune health centres, formed a non-user group.

The mMom intervention consists of two text message programs designed to support women’s health during pregnancy and new motherhood. The message content was developed using Mobile Alliance for Maternal Health (MAMA)^25^ templates and further adapted for local acceptability. The ethnographic fieldwork process indicated EMW were comfortable using Tiếng Việt (Vietnam’s national language) for the text messages. The first program targets prenatal care and healthy foetal development, while the second targets postpartum care and infant development. The mMom programs were linked to the HMIS system through unique identifiers for each participating woman. This ensured the messaging was linked to the stage of pregnancy and clinical interactions with the CHW could be monitored. The text messages, sent automatically two to three times weekly, aim to inform participants of appropriate actions, encourage their use of MNCH services and increase their awareness of risk factors. Based on the identified limitations of traditional IEC approaches in Dinh Hoa and current literature gaps, the mMom project added an active BCC component through messages with calls to action and two-way interaction with health staff. Within each program, IEC and reminder messages provide information and remind women to take critical actions, such as getting a tetanus immunization. Interactive messages request women to respond to monitoring questions, such as whether they have recently visited the health centre, and scanning messages link the interactive messages to the HMIS. In each program, 15% of messages require a response from the participant. If a response is absent or suggests a risk, the project software automatically informs that individual participant’s CHW, and further reminds CHWs to communicate regularly with the women under their care in a unique user-provider interaction model.

The intervention was run primarily by the CHWs. Across the 12 intervention user and non-user communes, 70% of CHWs were women and 81% had minority ethnicity. The intervention was managed by the Dinh Hoa District Health Centre (DDHC), and supervised by the TNHD. The mMom project was initiated on 1 July 2014 in Dinh Hoa, and had provided MNCH information via mHealth to 961 participant women by July 2017.

### Evaluation methods

Qualitative assessment of the project was necessarily constrained by the real time nature of the project and the resources available for evaluation. The MOH partners on the project imposed tight timelines for project reports in order to ensure the project results were included in scale up discussions for budget support at both the provincial and national levels. Although a pre- and post-intervention survey was administered with all participants, these data are still being analysed and will be published separately. The qualitative assessment of the project’s impact was based on mid-term and final assessment conducted in December 2014 and May 2016 by teams of semi-external evaluators who knew the project well but did not participate in implementation (evaluators from SFU, MOH, and local MNCH and IT experts, supported by staff translators from PHAD). The evaluation included document review; field observations; and focus group discussions and in-depth interviews with project stakeholders including TNHD and DDHD officials, CHWs, and intervention users and non-users. For the final evaluation, two focus groups and eight interviews were conducted with CHWs, and over 60 participant women (~15% of the sample) provided feedback through four focus groups and 30 interviews. A convenience sample of EMWs participating in the project was selected for focus groups and interviews at the time of visits to their villages based on their availability. The team continued to select women for participation in the evaluation until saturation of emerging themes was reached. Interview guides for the focus groups and in-depth interviews were developed with theoretically derived themes in mind. Interviews were digitally recorded and transcribed by PHAD staff. Each evaluator took extensive notes and assessed their notes to identify key themes. Evaluators met periodically during the interview and focus group process to compare notes and discuss emerging themes. Given the relatively small size of the project, qualitative software was deemed unnecessary.

## Results

Demographic characteristics of the project participants (both users and non-users) are described below. Given the stratified random selection of communes, and demographic similarity of the communes in this District there were no significant differences between users and non-users (Table 1) (Although this data suggests a high education level in the study population, this is consistent with the emphasis on education in Vietnam where the literacy rate is ~97% of the population. Moreover, targeted EMW include younger women who had better access to education than older EMW.)

**Table 1:** mMom participant demographic data

Demographic indicator	Percentage of project participants
Median participant age (years)	26
Completed secondary school	42.2%
Currently working in agriculture or local factories	66.6%
Ethnic background	
Minority ethnicity	75%
Kinh (majority) ethnicity	23.6%
Mobile phone usage	
Have their own mobile phone	91.4%
Share a phone with someone else	4.5%
Send and receive text messages	95%

### Acceptability

Participants were unanimous in their satisfaction with receiving regular, timely MNCH information via their mobile phones, asserting that this medium was highly convenient and allowed information to be saved and shared. When asked, most participants reported willingness to pay a nominal fee for the service, suggesting high perceived value of the intervention despite economic constraints. Further, most expressed desire to receive messages during future pregnancies:

During my next pregnancy, I would still continue to participate. Because I did not know much for my first pregnancy, I still want to understand more for my second child.—*P1, focus group, Lam Vy Intervention Commune*

Participants also appreciated the project’s electronic format, which helped to mitigate the sometimes high financial and logistical costs of travelling to a health centre.

My house is 18–20 km away from the hospital, very far, so it is difficult. Medicine doesn’t cost that much, just travel fees.—*23-Year-old Tay woman, with one 8-year-old and one 6-month baby.*

CHWs viewed the mMom intervention positively, reporting that it had improved their ability to monitor pregnancies and infant health, resulting in heightened efficiency and reduced workloads. In this way, the intervention was demonstrated to be highly acceptable to its most critical stakeholders.

### Increases in knowledge

All participants reported that the BCC and reminder messages provided valuable MNCH information, which promoted their knowledge of pregnancy and newborn care:

For my first child [pregnancy], I was not aware of many things: for example, during the first month of pregnancy, I have to take an iron supplement; at 6 and a half months, I have to take calcium […] but for this second child, the program reminds me, so I know how to take care of myself and my baby.—*P1, focus group, Binh Yen Intervention Commune*

When seeing the messages, I feel that the program is very beneficial, good for pregnant women, children; good for moms with small babies. The program has a lot of useful information that I have learned from to take care of my baby properly.—*27-Year-old Tay woman with one 5-year-old and one 6-month baby*

### Behaviour change communication

The intervention’s use of BCC was found to be more effective than traditional IEC approaches through increasing participants’ action-oriented behaviour and engagement with the health system. While traditional IEC methods for MNCH in Dinh Hoa included printed materials and monthly group counselling sessions, the low informative value and frequency of these services, in conjunction EMW’s poor education and marginalized status, had resulted in infrequent interaction between EMW and health workers. Prior to the intervention, several CHWs asserted that despite their presence at the health centre, pregnant women and mothers would rarely initiate a phone call or visit, nor would they report health problems until the issue had become an emergency. Post-intervention, CHWs expressed satisfaction with women’s increased active care-seeking through phone calls to ask questions and health centre visits:

[Before the project], if the child had fever or ate poorly, they would leave them home. Now they bring them to the health centre. The knowledge of participating mothers has also increased. For example, for immunization, the mothers are very concerned: they ask about infant vaccinations or how can they know what is injected in the appropriate week.—*Head of Commune Health Centre, Dong Thinh commune*

Participants themselves reported that the messages provided knowledge on what physical symptoms, at which times, warranted seeking medical attention:

[The project] sent messages telling me to pay attention—if I see something strange in my baby, then to take him to the clinic.*—30-year-old woman with infant under 1*

For example, the project texted me: when you were pregnant, did you have fever, feel sick, or have bleeding? I didn’t know about these problems before I was pregnant. Thanks to the messages, I know more.—*28-Year-old Tay woman with 3-year-old child, currently 7 months pregnant*

Several stated that the messages helped to distinguish between normal experiences (e.g. oedema, morning sickness) and potentially dangerous experiences (e.g. heavy bleeding, high fever) which required swift action by calling a CHW. One 31-year-old Tay participant stated that when facing recurrent stomachaches during her pregnancy, she visited the health centre because the mMom messages had warned specifically to seek help on that occurrence. In this way, the intervention’s action-oriented information helped participants to identify danger signs and seek care earlier, which in turn promoted CHWs’ ability to prevent and de-escalate problems in pregnancy, and to avoid severe complications.

### Husbands’ involvement in MNCH care

The mMom project had a meaningful impact on husbands’ engagement with MNCH care. Many women mentioned that they shared the messages with their husbands, and that they would discuss the message content together. Several stated that the messages should be sent to husbands as well:

Often fathers don’t know how to take care of babies, or when to take their wives for a pregnancy check-up. I am a woman, so I have to take the initiative—it would be better if fathers could also receive messages.—*P3, focus group discussion, Lam Vy Intervention Commune*

In interviews, the majority of husbands stated that they had learned new information from the mMom messages. Husbands’ stated interest in receiving the SMS messages themselves suggested that their interest in MNCH had increased:

Before planning to have a baby, I searched on the Internet and asked for advice from old people and parents. This program [mMom project], my wife told me about it, so we participated. Because this is our first child, we felt puzzled; we do not have many experiences; we felt worried; but receiving the messages from the project helps us know what to do, makes us feel better. The program reminds us of when to do what. My knowledge has increased a lot; for the second child, we will feel less bewildered.—*Husband, 32, to 26-year-old Nung woman with 11-month baby*

Of course [I would be willing to receive messages], both of us should receive the SMS so that we could both gain knowledge for taking care of our baby.—*Husband to 27-year-old Tay woman with 5-year-old and 6-month baby*

Yes, my husband reads the SMS [messages] too, he even reminded me to go for a vaccination when I forgot.—*27-Year-old Kinh woman with 5-month baby*

In a social context where maternal and infant health have traditionally been considered the domain of women, husbands’ increased interest and engagement in this area is a positive effect of the mMOM project.

### Strengthening relationships

The MNCH information in the mMom messages and the confidence it promoted amongst participants had the effect of increasing participants’ interaction with CHWs, and strengthening their relationships. The project helped to minimize anxiety amongst participants, who reported that knowing what to expect at each stage helped them to feel less worried throughout their pregnancy. This was particularly salient for participants pregnant with their first child. Women’s increased confidence in their own knowledge positively impacted their interactions with the health care system:

I’ve contacted the health staff when my baby has had diarrhoea or fever… I feel confident. I don’t feel hesitant to call the health staff.—*26-year-old Nung woman with 11-month baby*

For my first child, whenever I had a problem, I always went to the health centre, but now I just need to call the health staff’s phone number, it is more convenient. Yes, I feel good when I call the health staff, they are very enthusiastic.—*27-Year-old Kinh woman with 5-month baby*

CHWs also reported that the project had enhanced their relationships with local women. They asserted that women were phoning more frequently to ask MNCH-related questions, resulting in higher quantity and quality of interactions. CHWs also reported that the message content had an enabling effect on their readiness to initiate discussions:

Women ask more questions when they have more information from the text messages. They ask us right away when they have any questions.—*CHW 2, focus group*

We only need to input women’s phone numbers, then they receive messages right away. Most of the participants call the health staff if they have questions. Two-way interaction like that is very beneficial.—*CHW 1, focus group*

### Workload reduction for CHWs

Most CHWs expressed that the mMom intervention had reduced their workloads and improved their working efficiency. Many noted that because mMom was providing timely information to pregnant women, they had to spend less time attending to group counselling.

In the past, we have had to organize communication events a few times per year for pregnant women and women with small kids. But now that the SMS system is in operation, we don’t have to give group counseling.—*CHW 1, in focus group discussion*

Several CHWs stated that prior to the mMom project, they spent considerable time seeking out pregnant women and new mothers to provide them with care, but that mMom project participants had begun to actively seek care. Several remarked that the automatic sending of messages helped them to feel at ease, knowing that women were accessing the necessary MNCH information for a healthy pregnancy and early infanthood. Commune doctors also indicated they were able to focus more on their areas of work with the help of the CHWs and their increased capacity. In this way, the mMom project allowed for improved focus on priority areas of maternal and infant care.

#### Non-user group

Once being informed of intervention, women in the non-user group expressed high interest in mHealth. Many stated that MNCH information via text messaging would be very convenient:

I want to learn [about MNCH] but I don’t have time. If I could stay at home and receive messages via my mobile phone, it would be much more convenient.—*38-year-old woman with 19-month toddler and an older child, Trung Hoi non-user commune*

The non-user group also reported fewer opportunities to access information and interact with their CHW. Several non-participant women reported concern about not knowing which pregnancy or newborn health issues warranted medical care, and had low confidence in bringing questions to their CHW. Many reported a dearth of information:

There is no information [about vaccinations before pregnancy]. Here, sometimes we don’t really know when we are pregnant, not until being 1–2 months pregnant, when we feel something different. Many people here are like that.—*C1, focus group, Linh Thong non-user commune*

While non-participant women reported semi-regular contact with their CHW during pregnancy and new motherhood, they were less active in care-seeking during these critical periods:

Not really, I would only answer if they asked questions.—*C3, focus group, Linh Thong non-user commune, in response to ‘do you ask the health staff questions’*

## Discussion

### Main finding of this study

The mMom project’s most important impact was in strengthening relationships between women and their health providers. The mHealth messages were found to promote participants’ access to the health care system by providing important information which could be utilized as a starting point for discussions. The proactive provision of actionable MNCH education helped to bridge the interaction gap experienced by ethnic minority women as a result of marginalization, resulting in both CHWs and participants reporting that local women had gained confidence in actively seeking care.

mHealth holds great potential to promote equity in this context, because despite their lower social and economic status, most ethnic minority peoples in Vietnam use mobile phones. This suggests that effective mHealth interventions may disproportionately benefit historically underserved communities in this context, representing a powerful avenue to promote health equity for ethnic minority groups living in mountainous or remote regions in Vietnam.

### What is already known on this topic

Multiple studies in Asia and Latin America identified receptive attitudes towards mHealth projects, ranging from 73 to 99%^26–29^ even amongst rural and low literacy communities. This openness to mHealth was reflected in the mMom study, as participants’ were unanimous in their willingness to receive messages containing health information. The acceptance of mHealth amongst CHWs in the mMom project also mirrors literature suggesting that mobile health can support CHWs in managing a heavy workload, resource constraints and substandard tools to provide services.^30^

There is relatively little evidence on the effectiveness of BCC over IEC in mHealth interventions in LMICs, as most research has been conducted in high income settings or presents only descriptive studies without a strong evaluation component.^22^ However, a 2014 meta-analysis of mHealth MNCH projects reported that interventions based on behaviour change and motivational language were more likely to achieve their goals,^31^ and a 2017 New Zealand intervention aimed at Maori and other ethnic minority groups used BCC methods which were well received by participants.^32^ As recommendations from the literature on applying BCC in mHealth emphasize communicating in the desired language of end users, attending to cultural factors, employing two-way communication and connecting participants to existing health infrastructure,^22,31,32^ these areas were carefully considered in the mMom intervention.

### What this study adds

In this project context, mHealth and the specific application of BCC were more effective than traditional unidirectional IEC methods in supporting access to MNCH care, as the action-oriented message content promoted increased contact between community women and health providers. This was a key finding in a setting where EMW have been relatively left behind by development progress, continue to experience fewer interactions with health services, and face poorer health outcomes. The mMom messages acted as a mediator and dialogue-initiator between women and health workers, and the two-way communication enabled CHWs to follow up with participants more regularly. The mMom intervention demonstrated the impact of shifting beyond traditional IEC approaches, as the project effectively increased participants’ demand for quality natal care.

Few studies have examined the impact of mHealth on participants’ confidence, satisfaction with health care or relationships with health workers, but available literature has found text message interventions to support higher satisfaction with antenatal care, higher confidence and lower anxiety during pregnancy,^33^ increased empowerment to make informed decisions about health care, and increased knowledge in interactions with health workers.^34^ The mMom project found that providing actionable and motivational information promoted participants’ active participation in care-seeking, which influenced the frequency and quality of their health system interactions.

### Limitations of this study

This study was conducted within a contained geographical area, amongst ethnic minority populations who had contact with the health system, as CHWs introduced the project to women who presented at the health centre. As such, it’s possible that the project excluded members of the target population who were not known to health workers or linked to a health centre, due to extremely remote residence, poverty or language barriers.

## Conclusion

The project demonstrated the acceptability of mHealth for maternal health in a Vietnamese region with high mobile phone usage. mMom participants reported having developed stronger relationships with their CHWs, which has particular salience in a context where EMW have had relatively lower contact with the health care system and experience inequitable maternal and infant health outcomes. Enhanced relationships with care providers builds demand for accessible health care, which is relevant to policy decision-makers aiming to ensure an adequate supply of services based on demand. In this way, interventions such as mMom which stimulate demand for health services may play an important role in promoting sustained resource allocation to previously under-resourced environments, which is critical for continuing to bring about health equity for underserved populations. This suggests strong potential for mHealth application amongst populations who face structural and geographical marginalization, and for whom lack of MNCH information is a primary determinant of poor maternal health.
